# ‘Motivating Implicit Chinese to Express Themselves Is the Biggest Barrier’: A Qualitative Study of Chinese Researchers' Perceptions of Barriers and Facilitators to Patient Engagement in Research

**DOI:** 10.1111/hex.70112

**Published:** 2024-11-21

**Authors:** Lin Yang, Yu‐xiao Liu, Bi‐xia Wang, Meng‐jiao Yu, Wei‐Wei Bian, Cai‐feng Wang, Hong Ruan

**Affiliations:** ^1^ Department of Nursing, Shanghai Ninth People's Hospital Shanghai JiaoTong University School of Medicine Shanghai China; ^2^ School of Nursing Shanghai JiaoTong University Shanghai China; ^3^ School of Nursing Naval Medical University Shanghai China; ^4^ Shanghai Nursing Association Shanghai China

**Keywords:** barriers, China, culture, facilitators, patient engagement in research, qualitative research, researcher

## Abstract

**Background:**

Patient Engagement in Research (PER) has demonstrated benefits for patients, researchers and research outcomes. However, China lacks substantial experience in implementing PER. The implementation of PER in China faces unique challenges due to social‐cultural differences. This study explores the perspectives of Chinese researchers to identify barriers and facilitators, aiming to guide future PER initiatives and enhance the role of patients in research.

**Method:**

Purposive sampling was employed to recruit clinical researchers with diverse healthcare backgrounds in China. Semi‐structured interviews, conducted by a qualified researcher, followed interview guidelines derived from a literature review and pilot study modifications. Thematic analysis was applied using QSR Nvivo 8.0.

**Results:**

A total of 13 participants were included. Five main themes were identified from interview: (1) selection of patients for research engagement, (2) strategies to alleviate the patient burden in implementing PER, (3) strategies to encourage patients for active expression, (4) benefits to attract patient engagement and (5) researcher's preparation.

**Conclusion:**

The cultural trait of ‘reservedness’ in Chinese culture hinders active expression by patients in the research engagement process. Researchers tend to recruit patients with specific characteristics and emphasize the importance of aligning benefits with patient values to motivate engagement. Addressing patient burden is crucial, and researchers should be well‐prepared before PER. These findings underscore the necessity of adopting culturally adapted strategies in PER to effectively address specific challenges.

**Patient or Public Contribution:**

The public participated in the interpretation of the interview results, enriching our understanding of the results.

## 1 Introduction

In the field of medical research, Patient Engagement in Research (PER) has emerged as a highly scrutinized topic. PER refers to active, meaningful and collaborative interactions between patients and researchers at all stages of the research process. The formulation of research decisions involves contributions from patients as partners, requiring researchers to fully acknowledge their specific experiences, values and professional knowledge [1]. The application of PER has positive effects on research outcomes, researchers and participants alike. PER ensures the clinical value of research topics, transparency and legitimacy in the research process, and contributes to the translation of research results into practical applications [2]. For researchers, it enhances insights and understanding of the research field, reduces participant attrition, improves data collection tools and enhances research efficiency, demonstrating extensive practical value [3]. For patients, it promotes their understanding of more information about diseases and treatments, improves the trust relationship between researchers and patients, and has been reported to enhance the quality of life for patients in certain disease areas [3].

In nations like the United States and Canada, associations or research institutes dedicated to PER have been established to provide financial support for the development and application of PER, and have accumulated a wealth of experience and demonstrated successful cases [4, 5, 6]. Researchers have developed specialized frameworks that aim to direct the practice of patient‐engaged research and encourage cooperation between patients and researchers [7, 8, 9]. Despite the increasing recognition of the importance of patient engagement in research, there is currently a notable absence of research or practical experience reports in this field in China.

The promotion and application of PER face several barriers, especially when introduced in a new setting [10]. When bringing PER into China, it is essential to acknowledge significant differences in social culture, healthcare environments, and research contexts compared to other countries. These disparities may influence patients' attitudes toward PER and present distinctive barriers in implementing PER, necessitating the development of specific strategies to facilitate PER [11]. Therefore, gaining the perspectives of Chinese researchers on barriers and strategies in PER is of great significance. They possess direct patient interaction experience and an extensive understanding of China's culture and environment. Addressing challenges in PER is primarily the responsibility of researchers, making it logical and valuable to derive strategies from their perspective.

The objective of this study is to implement a qualitative research methodology to gain insight into Chinese researchers' perspectives on potential barriers to conducting PER in China and explore methods to facilitate PER, based on their experiences in previous clinical research. This study is expected to provide guidance for future PER in China and promote a more valuable role for patients in medical research and better medical outcomes.

## 2 Materials and Methods

### 2.1 Recruitment and Sampling

This study was approved by the Institutional Review Board at Shanghai Ninth People's Hospital. Informed consent was obtained from all individual participants included in the study. The purposive sampling method was employed to select participants. Interview subjects were recruited in Shanghai, China. Shanghai is one of the largest cities in China with rich resources in the healthcare field and most of the medical staff have experience in research, so recruiting qualified respondents from them is accessible. According to our research objectives, participants were required to have experience in leading or participating in clinical research, directly collecting patient data, or implementing interventions on patients, spanning both clinical medicine and nursing research fields. Researchers solely engaged in basic research, such as animal experiments, were excluded from consideration. Despite potential unfamiliarity with PER, Chinese researchers possess extensive experience in conducting research and interacting with patients in the local context, enabling them to offer valuable insights and recommendations regarding potential barriers and facilitators of PER. Given that Chinese clinical researchers usually work for hospitals or medical schools, participants in this study were recruited from healthcare professionals working in tertiary hospitals or community hospitals, and medical school educators. The inclusion criteria for the study subjects were as follows: (a) researchers had participated in clinical research projects within the past 5 years, involving direct contact with patients, including face‐to‐face questionnaires, interviews and interventions. (b) Bachelor's degree or above. The study's sample size was determined by reaching information saturation, indicating that no new topics or insights were emerging. After each participant interview, data analysis was conducted. If no new themes emerged during the analysis and after interviewing an additional participant, data saturation was considered achieved, and the interviews were concluded.

### 2.2 Semi‐Structured Interview

A phenomenological research paradigm was adopted. The manuscript adheres to the Consolidated Criteria for Reporting Qualitative Research (COREQ) checklist [12]. A female PhD student and orthopaedic nurse with experience in qualitative research, conducted semi‐structured, one‐on‐one in‐depth interviews. Before the interviews, participants were briefed on the study's purpose and significance. It was clarified that all interview materials would be securely stored and used exclusively for research purposes. To ensure confidentiality, participants' names were replaced with numerical codes. After obtaining informed consent, the interviews commenced with simultaneous audio recording. The interviews were scheduled at times convenient for the participants and conducted in quiet locations.

The interviews commenced with an exploration of researchers' awareness of patient contributions in their past research experiences. Subsequently, a unified and standardized introduction to the definition of PER was provided to ensure respondents had a correct perception of PER during subsequent interviews, mitigating the risk of any potential ineffectiveness due to a lack of comprehension. If respondents had any misunderstandings regarding the concept of PER, they were immediately addressed and clarified. Upon ensuring that respondents comprehended the concept of PER, subsequent interviews were initiated. The initial step involved querying respondents about their perspectives on the significance of PER. Open‐ended questions are then posed to explore potential barriers to PER implementation in China and to gather insights on how to address these challenges and promote PER. Previously, a review of barriers to PER had been conducted, summarizing the barriers as follows: (1) inadequate infrastructure for conducting PER, (2) barriers to establishing researcher–patient relationships and (3) obstacles in maintaining collaborative partnerships [13]. Part of the interview guideline is built upon these findings, giving rise to the probes within the interview outline. Specific questions based on the probes were developed to allow for in‐depth interviews with participants. A preinterview pilot study was conducted to refine the interview design. The finalized interview guideline is presented in the Appendix.

### 2.3 Data Analysis

After the conclusion of each interview, a nurse with a bachelor's degree transcribed the interview data. According to the research objectives, the inductive method is adopted to explore and generate new insights through interviews. Employing Braun and Clarke's reflexive thematic analysis process [14]. The iterative process of data collection and analysis was guided by constant comparison, ensuring a thorough examination of emerging themes. QSR Nvivo 8.0 Chinese version was employed as the software tool. The analysis began with careful reading and familiarization with the data to identify descriptive codes. These codes were then summarized into overarching coding patterns that provide the basis for identifying themes for analysis. Each theme underwent a meticulous process of review, refinement and naming to ensure logical coherence and, in particular, to reveal the barriers and facilitators to PER in China.

## 3 Results

**Figure 1:**
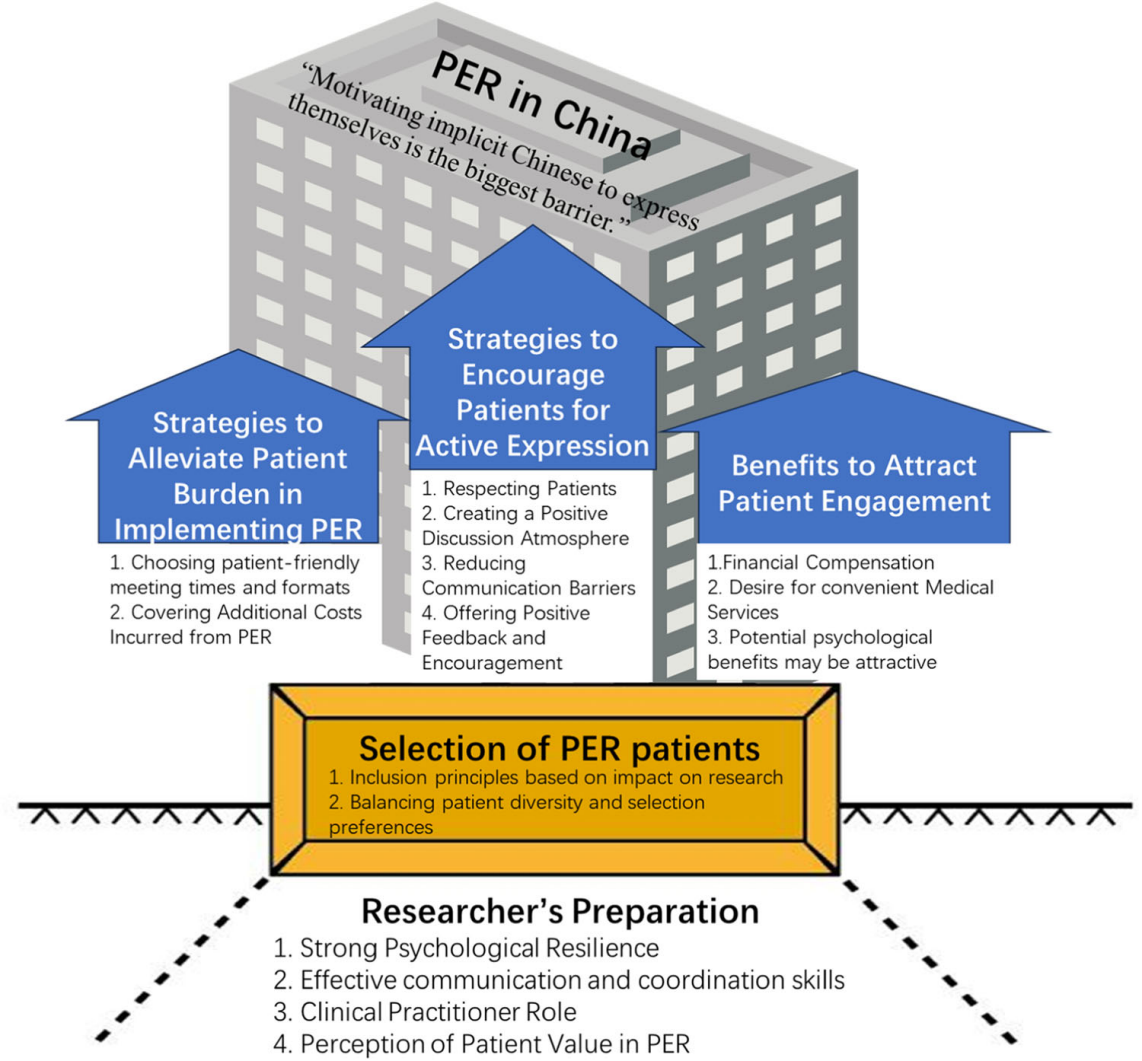
Relationship map between themes and sub‐themes.

A total of 13 participants were included, with demographic information as shown in Table 1. Five participants (D1–D5) were from the medical research field, and eight (N1–N8) were from the nursing research field. Five main themes and 15 sub‐themes were extracted (Table 2). Relationships between topics were mapped (Figure 1).

**Table 1:** Interview sample demographics (*n* = 13).

Participate	Sex(F/M)	Age (years)	Work experience (years)	Education level	Position	Professional title	Research area	Workplace
D1	M	34	8	PhD	Clinical Physician	Associate CP	Orthopaedics	TH
D2	F	40	18	PhD	Clinical Physician	Associate CP	Plastic Surgery	TH
D3	M	43	16	PhD	Department Head	CP	Orthopaedics	TH
D4	F	30	6	Master's	General Practitioner	Attending Physician	Traditional Chinese Medicine	CH
D5	F	29	6	BS	General Practitioner	Attending Physician	Internal Medicine	CH
N1	F	30	5	Master's	Teacher	Lecturer	Paediatric Nursing	Specialized College
N2	F	29	5	Master's	Nurse	NI	Cardiology Nursing	TH
N3	F	34	13	BS	Nurse	Supervising NI	Orthopaedic Nursing	TH
N4	F	33	10	BS	Head Nurse	Supervising NI	Plastic Surgery Nursing	TH
N5	F	34	11	Master's	Head Nurse	Supervising NI	Oral and Maxillofacial Surgery Nursing	TH
N6	F	29	5	Master's	Clinical Nurse	NI	Orthopaedic Nursing	TH
N7	F	43	24	Master's	Deputy Director of Nursing Department	Chief Nursing Officer	Nursing Management	TH
N8	F	42	20	PhD	Teacher	Associate Professor	Anaesthesia Nursing	Medical School

Abbreviations: BS, Bachelor of Science; CH, Community Hospital; CP, Chief Physician; F, female; NI, Nursing Instructor; PhD, Doctor of Philosophy; TH, tertiary hospital.

**Table 2:** Themes and sub‐themes emerged from qualitative research.

**Main Themes**	1. Selection of Patients for Research Engagement	2. Strategies to Alleviate Patient Burden in Implementing PER	3. Strategies to Encourage Patients for Active Expression	4. Benefits to Attract Patient Engagement	5. Researcher's Preparation
**Sub‐themes**	1. Inclusion principles based on impact on research 2. Balancing patient diversity and selection preferences	1. Choosing patient‐friendly meeting times and formats 2. Covering Additional Costs Incurred from PER	1. Respecting Patients 2. Creating a Positive Discussion Atmosphere 3. Reducing Communication Barriers 4. Offering Positive Feedback and Encouragement	1. Financial Compensation 2. Desire for convenient Medical Services 3. Potential psychological benefits may be attractive	1. Strong Psychological Resilience 2. Effective communication and coordination skills 3. Clinical Practitioner Role 4. Perception of Patient Value in PER

### 3.1 Theme 1: Selection of Patients for Research Engagement

#### 3.1.1 Inclusion Principles Based on Impact on Results

Participants emphasized the importance of first considering whether the selection of patients will have an impact on the results of the research and that the selection of specific characteristics of patients that would lead to a large bias in the results should be avoided. The scientific validity of the study was considered to be the primary.

One thing to consider is whether education background might bias your results. If it doesn't, then go ahead and choose participants with a good education background. But if it does, then you really need to make sure the education background is uniform. (D2)

#### 3.1.2 Balancing Patient Diversity and Selection Preferences

Participants emphasized the need for diversity in patient selection, including various age groups, cultural backgrounds and education levels. This ensures a comprehensive representation of patient needs and perspectives, enhancing the overall inclusivity and representativeness of research results.

I think we should purposefully pick a diverse group, like we do in interviews. Choose people from different levels, cultural backgrounds, and age ranges to participate. (N8)

However, some participants mentioned that patients with different characteristics may behave very differently at PER. Engagement can be difficult for some patients. Participants mentioned that in higher education, younger people may have a better understanding, and outgoing people are more likely to express themselves and can provide more substantive feedback. Patients with plenty of free time, those who need long‐term treatment or currently lack effective treatment options are more likely to be willing to actively engage in research

If the patient is older or has a lower level of education, it may be very difficult for them to understand your questions. They may not be able to provide many constructive opinions, or they might simply say, ‘I don't know’ or ‘I can't,’ rejecting you. (N6)

Some patients are still working, or need to take care of children at home, and life is quite busy. They might not be very likely to engage in your research… From my experience with bone tumor patients, and generally the disease is detected early, so they will be very interested in knowing whether there are any new treatments to extend life and reduce pain a bit. (D3)

### 3.2 Theme 2: Strategies to Alleviate Patient Burden in Implementing PER

#### 3.2.1 Choosing Patient‐Friendly Meeting Times and Formats

Participants suggested considering scheduling research meetings during their planned hospital follow‐up appointments to reduce their additional appointment burden. Participants mentioned that online participation may be an effective way to reduce time and transportation costs for patients so that patients can comfortably participate from home or workplace, avoiding the hassles of going out, which might enhance their willingness to participate.

When they come in for their follow‐up, like, if they have one every month, just schedule the meeting for that time. It might make things easier. (N1)

Try to utilize online methods so they don't have to come in person every time. (D5)

#### 3.2.2 Covering Additional Costs Incurred from PER

Participants indicated that travel and accommodation costs incurred by patients in the process of participating in PER need to be paid for by the researcher to reduce the financial burden of patients in participating in research.

Try to address or minimize the obstacles to their participation in the research. For instance, if you need them to come and taking the bus is inconvenient, cover the taxi fare for them. (N3)

### 3.3 Theme 3: Strategies to Encourage Patients for Active Expression

#### 3.3.1 Respecting Patients

Participants emphasized that maintaining respect for patients is a crucial factor in establishing and sustaining the relationship between researchers and patients. This is demonstrated through actively listening to patients' opinions, considering things from their perspective, understanding why they hold certain views, caring about their needs and feelings, engaging in empathy and providing information in an egalitarian manner to assist them in participating in the research, thus avoiding any sense of superiority.

Equality. It's not a pretend kind of equality, but a genuine one. Their opinions aren't just casually listened and brushed aside; instead, heard them and seriously consider why they think that way, valuing their viewpoints… It's essential to discuss the meeting arrangements in advance, putting ourselves in their shoes. (N1)

Show some respect, lower your stance. Don't live high and look down, like you're a teacher lecturing them. (D3)

#### 3.3.2 Creating a Positive Discussion Atmosphere

Participants highlighted the crucial importance of creating a positive and participative atmosphere during PER meetings. However, due to the modest and implicit characteristics of Chinese patients, it may be challenging to foster such an environment. To address this, measures such as training researchers to facilitate open discussions, ensuring patients feel comfortable and unrestricted, implementing turn‐taking for speaking and providing anonymous channels for opinion sharing were suggested. These steps aim to encourage active participation and overcome cultural barriers to open communication.

Chinese people are more implicit, it is very likely that the situation is that everyone does not speak, which affects the effect of this engagement. I believe this would be the most difficult challenge, need a researcher or a lively person to mobilize the atmosphere. (N7)

When the atmosphere is active, then everyone will speak up, you can go to name some of those who did not speak (patients) to ask if there are any ideas… if they really do not speak, so that he will finally write out these comments. (N2)

#### 3.3.3 Reducing Communication Barriers

Participants thought that the reduction of communication barriers depends mainly on the researcher's adjustments and improvements. It was suggested that researchers should respect the differences in communication due to the diversity of patients, explain in language that patients can easily understand, not use jargon and explain multiple times to ensure that the message is conveyed smoothly.

Don't talk too much medical, research jargon, then the patient won't understand for sure…talk a few times, with words that he can understand. (D1)

#### 3.3.4 Offering Positive Feedback and Encouragement

Participants emphasized the importance of providing positive feedback to patients' suggestions. This feedback serves not only as recognition and gratitude for the patients' contributions but also to stimulate their enthusiasm for participation. In terms of content, it is crucial to affirm the significant value of the viewpoints presented by patients. In cases where patients' opinions are not adopted, it is essential to honestly delineate the reasons for nonacceptance and explain the rationale behind selecting specific approaches. Some respondents noted that individual feedback might not be necessary in large‐scale studies, but in situations with a smaller number of participants, providing appropriate explanations is deemed necessary.

Express things verbally or, if possible, throw in some material rewards – even a certificate can work. Since everyone's desires vary, it's crucial to figure out what each participant wants or why they engage at first. Then, tailor your encouragement methods based on these motivations. (N3)

Just be honest about why a suggestion wasn't taken and why the chosen approach was better. Thank them for their contribution. It's as simple as that. (N2)

I don't think feedback is that necessary (on why their suggestion wasn't adopted), especially if there are a lot of patients involved. No need to explain everything individually. But if it's just a few people, then yeah, you can explain a bit. (D1)

### 3.4 Theme 4: Benefits to Attract Patient Engagement

#### 3.4.1 Financial Compensation

Participants indicated that financial compensation plays a crucial role in attracting patient engagement and that Chinese patients may prioritize tangible economic benefits rather than solely being driven by personal interest or the perceived value of engaging in research.

Foreigners might think, ‘I'm treating engaging in research as a big thing, I'm passionately committed and find it very valuable.’ But I believe people in China are more concerned about the financial return. (D1)

Offer them some decent compensation, and of course, it has to be a good amount. Honestly, nowadays, if you give them two hundred bucks (RMB), they might not even want to come. (N5)

#### 3.4.2 Desire for Convenient Medical Services

The participants mentioned that patients may desire closer contact with their doctors through PER to access convenient medical services, such as easy appointments with specialists, especially in cases where chronic patients require ongoing medical support.

For example, when scheduling appointments with specialists, provide them with some conveniences… They can contact the doctor more easily. (N3)

#### 3.4.3 Potential Psychological Benefits May Be Attractive

Participants felt that the process of patient's engaging in research provides an opportunity to release internal stress and receive psychological support. These benefits might serve as incentives to attract patient engagement.

When patients recall their experiences of treating illnesses, it often helps them release some of the pressure they carry within. (N1)

### 3.5 Theme 5: Researcher's Preparation

#### 3.5.1 Strong Psychological Resilience

Participants emphasized that researchers need the courage to face setbacks during the implementation process, maintaining the courage and enthusiasm to continue without giving up easily. Researchers need to prepare themselves for strong psychological resilience.

Dealing with all sorts of patients means dealing with a bunch of different issues, which can be pretty tough on the mind. (N2)

The one handling this job needs to be tough, not afraid of setbacks or getting no response, and even be thick‐skinned – perhaps needing to ask multiple times. They need to have strong mindset, be sociable, and not fear rejection. (N3)

#### 3.5.2 Effective Communication and Coordination Skills

Participants indicated that the researcher's communication and coordination skills are essential, not only for smooth communication with the research team but also for effective communication with patients as well, keeping the information flowing and transparent.

Effective communication and coordination skills are crucial because you need to connect with the research team and patients, and handle various situations… When communicating with patients, it's important to avoid a tone that may come off as too aggressive, considering that everyone has different perspectives, right? Get a few people to take a look and see how it reads. (N3)

#### 3.5.3 Clinical Practitioner Role

Participants felt that researchers with clinical work experience may be more advantageous, especially for establishing a deep connection with patients. Researchers with a clinical background may be more likely to develop a sense of closeness and trust with patients, which can be very helpful in the conduct of research.

I think having experience in clinical work might be more beneficial, you know, especially in establishing a closer connection with patients. (N8)

#### 3.5.4 Perception of Patient Value in PER

Many participants indicated that patient engagement can contribute to problem formulation and research direction, rather than just providing data. By sharing their actual experiences with the disease, patients bring the focus of the research closer to real needs. Perspectives from patients' viewpoints can transcend the preset boundaries of researchers, expanding the dimensions of research questions. Additionally, some respondents pointed out that the extent of patient involvement and influence varies at different stages of the research. During the phases of data analysis and research design, the predominant role is still played by researchers possessing professional knowledge.

Patient engagement may skew the research towards what they are really concerned about. (N8)

The parents (of the child) they are going to worry about more than I expected, not only about the disease, but also about the children's development, their intelligence in the future. (N1)

## 4 Discussions

In this study, participates indicated that the principles of patient selection were influenced by the topic and design of the study. When the study focuses on physiologic parameters (e.g., blood pressure, blood glucose, etc.), these indicators are usually not affected by factors such as level of education, free time, etc. Whereas, when studies have addressed cognitive or psychologically related factors (e.g., satisfaction, expectations, health literacy, etc.), which may be perceived differently by patients with different demographics, the representation of diverse patients should be given more consideration. Interviewees referred to the significance of patient diversity, following the concept of PER, the inclusion of patients aims to capture a broader range of voices. Only by fully considering this diversity can the comprehensiveness, reliability and effectiveness of the research be ensured [15]. However, respondents also mentioned that certain demographics might encounter significant barriers in PER. Previous studies have reported fewer barriers in engaging certain patient characteristics in research, such as younger age, higher education, extroverted personality and patients with more available time [16, 17]. An interview with homebound older adults and caregivers in the United States indicated that barriers to participation in research existed in addition to time constraints and caregiving responsibilities, personal physical barriers and also fear of lack of contributing skills or expertise [18]. Therefore, the fact that there are greater barriers to implementing PER in some specific groups of patients may exist, which poses a challenge to achieving population diversity.

Given the extremely limited experience of Chinese researchers in PER at present and the lack of corresponding policies, a reasonable strategy for initial attempts is to reduce the difficulty of implementation. Without compromising the results of the study, it is possible to collaborate with a ‘better’ patient to help researchers get familiar with the operational procedures of PER, accumulate experience and gradually enhance the feasibility of implementation [19]. This can aid researchers in their growth and promote the application of PER in China. When there is a preference for certain patients during selection, researchers should explicitly acknowledge the limitations of sample characteristics in the results analysis and conclusion sections, carefully interpreting the study findings. As experience accumulates and methods evolve, Chinese researchers can progressively expand the participant groups in PER, applying it to a broader range of diseases and research topics.

To alleviate the burden on patients during their engagement in research, respondents suggested choosing meeting locations convenient for patients. They recommended scheduling research meetings during patients' planned follow‐up appointments at the hospital. This strategy saves patients' time and transportation costs, making it highly feasible and recommended. Patients with severe or rare diseases in China are often treated in hospitals across regions in search of better outcomes. These patients may incur additional costs such as travel and accommodation fees when participating in research. It is recommended that researchers cover these expenses to alleviate the burden on patients, a conclusion drawn from the practical experiences of other scholars [20]. For patients who are nonlocal or have difficulty in travelling, respondents suggested that online forms of PER may be a good option that breaks through geographic constraints and offers more convenient scheduling, adapts to the patient's daily life and reduces the cost of PER. Discussing a convenient meeting format with patients was also seen as a sign of respect for patients [15]. However, online meetings have certain requirements for network configuration and devices, and some patients may find them challenging to operate. Additionally, there may be concerns about network security and data privacy, necessitating enhanced protective measures. Exploring whether there are differences in patient contributions between online and offline PER implementation, as well as investigating the experiences of both researchers and patients, is a direction for future research.

In this study, respondents emphasized the importance of respecting patients as the foundation for conducting any activities. Respecting patients and ensuring equal rights for all members are considered by many scholars to be the most crucial principles in PER [10, 21, 22]. Respectful collaboration is is defined by the Canadian Institutes for Health Research as ‘researchers, practitioners and patients acknowledge and value each other's expertise and experiential knowledge’ [23]. which means that respect involves not only demonstrating polite language and behaviour towards individuals but also respecting their contributions. It entails empathy, understanding their perspectives and genuinely incorporating their ideas into the considerations of the research. A proposal for how academic researchers can best collaborate with patients and community members on health research projects was constructed by Canadian researchers: roles and responsibilities, respectful collaboration, communication and planning and sharing knowledge [24]. Participants in our research also emphasized that minimizing communication barriers is a sign of respect and provides the foundation for facilitating patient expression. Efforts to enhance the quality of communication should involve avoiding the use of jargon that disregards the patient's cognitive level [21].

Participates indicated that one of the biggest barriers to implementing PER in China may be the establishment of an enthusiastic and expressive atmosphere in meetings for patients to speak, since Chinese people are subtle, introverted and have a low willingness to express themselves, which is greatly influenced by China's cultural and educational background. First, traditional Confucian values in Chinese culture emphasize humility, caution and self‐restraint, prioritizing collective interests over individual, which influence people's modes of expression, being subtle and euphemistic, and often reluctant to express views in public that are not in line with the majority or the authority. The history of Western countries has witnessed the Enlightenment, which may have contributed to their culture placing greater emphasis on individual rights and freedom of expression.

Secondly, for a long time in the past, Chinese education has focused more on memorization and the transmission of knowledge, and the pursuit of the ‘right answer’, so people believe that they should give the ‘right answer’ when asked. In addition, Chinese individuals place great importance on ‘face’, a desire to present a positive image and social status to the outside. They set high expectations for their performance and are acutely attentive to external evaluations, seeking approval and admiration. However, a lack of confidence in engaging in the challenging activity of PER and uncertainty about expressing the ‘right answer’, there's a fear of losing face if their contributions are ‘incorrect’. This uncertainty often leads individuals to refrain from speaking up. The emphasis on face has also brought a little benefit to PER, because refusing someone may be seen as not giving them a face, patients are embarrassed to refuse an invitation from a researcher in the role of their doctor. This dynamic could make it seemingly easier to invite patients to join research in China, but they may only superficially agree to take part, and will not be truly committed and outspoken in expressing their own viewpoints.

To address this barrier, respondents suggested informing in advance that patients' viewpoints will not be considered ‘incorrect’ or subject to criticism, incorporating practices like taking turns to speak during meetings and providing a chance for introverted individuals to express opinions anonymously [21]. Researchers should seek to understand patients' perspectives and engage in constructive discussions at appropriate times. Achieving these shifts in perception and fostering an inclusive atmosphere may require training for both the research team and patients. Researchers should invest time in building relationships, demonstrating genuine interest in participants' perspectives, and creating a supportive and non‐judgmental environment for open dialogue. Also, researchers should be mindful of participants' concerns about ‘face’ and social status. Respecting participants' dignity and autonomy, and acknowledging their contributions publicly can help alleviate concerns about losing face and encourage active participation.

In previous lessons from PER, negative emotions were expressed by patients when their contributions did not lead to changes in research decisions [20]. However, respondents in this study held a neutral stance on this issue, which may be attributed to the fact that Chinese patients' participation in PER is not driven by the pursuit of individual values. Their humble qualities may result in lower expectations regarding their ability to make significant contributions, so feelings of frustration are not overly strong when contributions are limited. Nevertheless, providing positive feedback on patients' expressions remains helpful in boosting their enthusiasm.

In this study, respondents believed that patients should be provided with incentives to engage in research, among which a substantial financial reward might be the most effective. A review indicated that 35% of PER researchers reported offering financial compensation and reimbursement for patients' time, knowledge and expenses during their engagement [10]. Our participates were contacted with Chinese patients of old age, who grew up in a time of scarce resources and economic hardship, so they expect financial rewards to reflect their engagement. Most of these patients have only an elementary school education or less, so it is often difficult to understand the value of their engagement with scientific knowledge and medical advances. This phenomenon reflects the economic and cultural perceptions of a particular age group in China. In PER practices in low‐ to middle‐income countries, it is also observed the need to compensate patients for the loss of income incurred due to participating in meetings [11]. An appropriate compensation policy needs to be carefully considered in the future to balance patients' contributions and rewards. Over the past decade, as more Chinese individuals pursue higher education, their engagement in scientific research has increased. They now value factors like social recognition, knowledge acquisition and self‐fulfilment over purely economic incentives. To meet diverse patient needs, personalized reward systems should be designed, considering individual preferences and expectations. This approach may better align with their values and enhance motivation for active participation. In previous research reports, young individuals have shown a preference for formal recognition of their contributions through certificates or recommendation letters [25].

In this study, participants expressed the belief that patients may be inclined to participate in PER with the expectation of obtaining more convenient medical services, as difficulties in booking appointments with specialists and long waiting times for medical treatment remain issues in China. However, this perception may be misguided, as researchers should not offer different medical services based on whether patients choose to engage in research or not, according to ethical principles. This highlights the need for more public education on PER for patients, enabling them to have a transparent understanding of what PER entails for them. Additionally, researchers require more training on ethical considerations to avoid ethical violations.

Some respondents believed that PER holds potential positive psychological effects. However, past experiences with PER have revealed negative psychological impacts, as some studies may require patients to recall unpleasant experiences [15]. While informing patients of potential positive psychological effects may attract their participation, it may also lead to heightened expectations, resulting in a mismatch between expectations and reality and leading to a negative experience. Therefore, it is essential to neutrally introduce the process to patients before recruitment, rather than resorting to false advertising. Researchers also need training to recognize this aspect. Several practices may help to minimize this traumatization of patients, such as incorporating a trauma‐informed intersectional analysis within the development of training, practice and evaluation with regard to public involvement in health research [26].

Respondents emphasized that PER researchers need to be prepared in various aspects. Researchers with strong psychological resilience can better handle potential setbacks, persist with patience and effectively solve problems, which has been unnoticed in previous studies. Additionally, excellent communication and coordination skills are crucial for ensuring efficient teamwork within the research team and fostering smooth communication with patients. Researchers with a clinical background find it easier to comprehend patients, establishing a sense of closeness and trust. Therefore, researchers need comprehensive preparation [27]. Researchers possessing these abilities can be given priority, and improve their readiness through assessment and training.

Respondents expressed an understanding of the value of patient involvement in research. Previous research has highlighted the importance of providing training and education to research teams. Insufficient understanding of the principles and implementation methods of PER can significantly impede the formation of an atmosphere that respects patients' contributions. Therefore, it is essential to conduct PER training for all team members [10, 21]. The participates suggested that patients may only provide value in certain stages of the research, which might be limited because the concept of PER has not been widely promoted in China. Some theoretical frameworks have been constructed to guide the design and implementation of PER, allowing researchers to choose based on the research type and feasibility of implementation [7, 8, 28]. The application of certain tools can also ensure the quality of PER. For instance, Julia Abelson et al. [29] developed the Public and Patient Engagement Evaluation Tool (PPEET) to assess the level of engagement of participants, projects and organizations. Clayton Hamilton et al. [30] created the Patient Engagement In Research Scale (PERIS), which allows patients to self‐assess their level of involvement in research. Our research team is currently working on translating these tools into Chinese, laying the foundation for PER implementation in China.

## 5 Limitations

This study has several limitations. First, the sample is specific to a particular region in China. Given the geographical and cultural differences across the country, strategies for promoting PER may vary. Our sample includes participants from various hospitals and academic institutions, covering different types of facilities and involving teachers from different regions, this diversity may help mitigate the limitations to some extent. In addition, the interviewer already had some research experience in the field of PER so that the impact of these perceptions and own research experiences were brought into the data analysis. To minimize this impact, interviewer received training on qualitative research principles, and the research team engaged in in‐depth discussions during the analysis stage to ensure the objectivity and credibility of the results.

## 6 Conclusions

PER has become a topic of great interest globally. The large patient population in China and the cultural inclusiveness provide a bright prospect for the implementation of PER in the country. This study delves into strategies for promoting PER from the perspective of researchers, exploring ways to encourage reserved Chinese patients to express themselves, personalize incentives to attract patient participation, and ensure thorough preparation by researchers. These suggestions not only offer practical methods for Chinese researchers but also provide valuable insights for researchers in other nations, contributing to the promotion of PER in different cultural and social contexts and offering solutions for the challenges of symbolic engagement. In the future, we aim to investigate barriers and facilitators of PER from the perspective of Chinese patients, further advancing the development of PER in China.

## Author Contributions

**Lin Yang:** writing–original draft, formal analysis, project administration, data curation, methodology, software, funding acquisition, investigation, visualization, resources. **Yu‐xiao Liu:** writing–review and editing, supervision. **Bi‐xia Wang:** formal analysis. **Meng‐jiao Yu:** formal analysis. **Wei‐Wei Bian:** writing–review and editing, supervision, formal analysis. **Cai‐feng Wang:** writing–review and editing, supervision, formal analysis. **Hong Ruan:** writing–review and editing, formal analysis, supervision, methodology.

## Conflicts of Interest

The authors declare no conflicts of interest.

## Supporting information

Supporting information.

## Data Availability

The data sets generated and analysed during the current study are available from the corresponding author upon reasonable request.
