# Supplementary material for: ‘Motivating Implicit Chinese to Express Themselves Is the Biggest Barrier’: A Qualitative Study of Chinese Researchers' Perceptions of Barriers and Facilitators to Patient Engagement in Research
Source: Health Expect. 2024 Nov 21;27(6):e70112. doi: 10.1111/hex.70112 (PMC11581954; doi:10.1111/hex.70112)
Supplement: Supplementary file 1 — Supporting information. [file HEX-27-e70112-s001.docx]

**Appendix**

| **Interview Guideline** | | |
| --- | --- | --- |
| **Introductory question:** Based on your past clinical research experience, what value do you think patients have brought to the research? | | |
| **Introduce the concept of PER:** The active, meaningful, and collaborative interaction between patients and researchers across all stages of the research process, where research decision making is guided by patients’ contributions as partners, recognizing their specific experiences, values, and expertise. | | |
| **No.** | **Interview questions** | **Interview topic** |
| 1 | Do you consider PER important? Why? In which stages of research do you think patients play a significant role? | Researcher's perception of the importance of PER |
| 2 | What obstacles do you think Chinese patients might face when engaging in research? | Extensive inquiry into barriers |
| 3 | For each barrier, what measures or methods do you suggest to address them? | Extensive inquiry into facilitators |
| **Transition phrases:** Here are some challenges mentioned in past studies. Think about whether these issues might occur in China's PER, and if they do, share your thoughts on how to tackle them. | | |
| 4 | What kind of patients do you think would be a good fit for engagement in research? | Recruit patients |
| 5 | What kind of relationship should be established between researchers and patients, how should it be established, and how can team cohesion be fostered? | Partnerships, team cohesion |
| 6 | Are there possible communication barriers between researchers and patients (especially those from different backgrounds)? What are your suggestions? | Communication barriers |
| 7 | Considering that patients may need to invest a significant amount of time to participate in research, do you think this is a possibility. If so, how can this burden be reduced without compromising the overall quality of the study? | Burdens associated with participation |
| 8 | What methods do you think can ensure patients make effective contributions to the research? If patients are not actively involved and hesitant to express their opinions, how do you suggest motivating them? | Patient contributions, avoiding symbolic involvement |
| 9 | When a patient's opinions are not ultimately adopted, how do you recommend communicating with that patient? | Addressing issues when patient opinions are not adopted |
| 10 | What challenges do you think PER poses for researchers? How can these challenges be addressed? | Challenges for researchers |
